# Sample size considerations for trials using cerebral white matter hyperintensity progression as an intermediate outcome at 1 year after mild stroke: results of a prospective cohort study

**DOI:** 10.1186/s13063-017-1825-7

**Published:** 2017-02-21

**Authors:** Francesca M. Chappell, Maria del Carmen Valdés Hernández, Stephen D. Makin, Kirsten Shuler, Eleni Sakka, Martin S. Dennis, Paul A. Armitage, Susana Muñoz Maniega, Joanna M. Wardlaw

**Affiliations:** 1 Neuroimaging Sciences, Centre for Clinical Brain Sciences (CCBS) FU303e, The University of Edinburgh, Chancellor’s Building, 49 Little France Crescent, Edinburgh, EH16 4SB UK; 2 Academic Unit of Radiology, University of Sheffield, C Floor, Royal Hallamshire Hospital, Sheffield, S10 2JF UK

**Keywords:** White matter hyperintensities, Sample size calculation, Study design

## Abstract

**Background:**

White matter hyperintensities (WMHs) are commonly seen on in brain imaging and are associated with stroke and cognitive decline. Therefore, they may provide a relevant intermediate outcome in clinical trials. WMH can be measured as a volume or visually on the Fazekas scale. We investigated predictors of WMH progression and design of efficient studies using WMH volume and Fazekas score as an intermediate outcome.

**Methods:**

We prospectively recruited 264 patients with mild ischaemic stroke and measured WMH volume, Fazekas score, age and cardiovascular risk factors at baseline and 1 year. We modelled predictors of WMH burden at 1 year and used the results in sample size calculations for hypothetical randomised controlled trials with different analysis plans and lengths of follow-up.

**Results:**

Follow-up WMH volume was predicted by baseline WMH: a 0.73-ml (95% CI 0.65–0.80, *p* < 0.0001) increase per 1-ml baseline volume increment, and a 2.93-ml increase (95% CI 1.76–4.10, *p* < 0.0001) per point on the Fazekas scale. Using a mean difference of 1 ml in WMH volume between treatment groups, 80% power and 5% alpha, adjusting for all predictors and 2-year follow-up produced the smallest sample size (*n* = 642). Other study designs produced samples sizes from 2054 to 21,270. Sample size calculations using Fazekas score as an outcome with the same power and alpha, as well as an OR corresponding to a 1-ml difference, were sensitive to assumptions and ranged from 2504 to 18,886.

**Conclusions:**

Baseline WMH volume and Fazekas score predicted follow-up WMH volume. Study size was smallest using volumes and longer-term follow-up, but this must be balanced against resources required to measure volumes versus Fazekas scores, bias due to dropout and scanner drift. Samples sizes based on Fazekas scores may be best estimated with simulation studies.

## Background

White matter hyperintensities (WMHs) are lesions in the white matter of the brain visible on magnetic resonance imaging (MRI). They are common and associated with stroke [1, 2], dementia [3, 4] and physical disability [5]. There is increasing interest in using WMH as an outcome in trials investigating cerebral small vessel disease and associated diseases such as stroke and dementia [4]. However, the mechanism for the development of WMH is not fully understood, and research, including randomised controlled trials (RCTs), into the role of modifiable risk factors such as hypertension, diabetes, smoking and hyperlipidaemia is ongoing [6].

Measurement of WMH volumes is not trivial, and there is not yet a consensus on how it should be done [7]. It is labour- and resource-intensive, requiring much time from staff with specialist image analysis skills. Fazekas scores are much easier to obtain as they may be read from brain scans within a few minutes by individuals with appropriate training. The Fazekas scale gives scores ranging from 0 to 3 each for periventricular white matter and deep white matter, which can be summed to give a total score from 0 to 6, with lower scores indicating less WMH burden.

Any trial using WMH as an outcome should adjust for important predictor variables such as baseline WMH and age. Also, prediction of WMH for an individual patient is difficult because there is much variability in WMH progression between patients [8]. However, previously published WMH sample size calculations have used unadjusted estimates [9, 10]. Therefore, we studied the predictors of WMH in a high-risk population, namely patients with mild stroke, in whom WMHs are an adverse prognostic marker [11, 12] and who would therefore benefit from interventions to reduce WMH progression. We examined ways, using study design, covariate adjustment and statistical analysis, to reduce sample size for clinical trials of WMH progression. We present our results on predictors of WMH and sample size calculations for hypothetical RCTs.

## Methods

### Patients and recruitment

We prospectively recruited consecutive patients aged over 18 years with a recent first symptomatic mild ischaemic lacunar or cortical stroke who presented to our hospital stroke service. All strokes were non-disabling, not requiring hospitalisation, and 98% of patients had scores of 4 or less on the National Institutes of Health Stroke Scale [13]. Patients with stroke due to genetic causes or dissection of the carotid or vertebral artery were excluded. The final diagnosis of stroke was reached as described below. All patients were able to give consent and undergo MRI at presentation. Patients with severe stroke, MRI contraindications or haemorrhagic stroke were excluded. All participants involved in the study gave written informed consent, and the study was approved by the Lothian Research Ethics Committee (reference 09/S1101/54).

### Assessments

Demographic and clinical history and examination data were collected at presentation by a trained stroke physician. Blood pressure was measured with clinic sphygmomanometers. Patients underwent carotid imaging, blood tests and other investigations as required for stroke.

Patients underwent diagnostic brain MRI at presentation on a 1.5-Tesla MRI scanner (Signa HDxt; GE Healthcare, Milwaukee, WI, USA) using an 8-channel phased-array head coil. Diagnostic MRI scans acquired at presentation included axial T2-weighted (repetition time [TR]/[TE] 6000/90 ms, 24 × 24-cm field of view [FoV], 384 × 384 periodically rotated overlapping parallel lines with enhanced reconstruction acquisition, 1.5 averages, 28 × 5-mm slices, 1-mm slice gap), axial fluid-attenuated inversion recovery (TR/TE/inversion time [TI] 9000/153/2200 ms, 24 × 24 cm FoV, 384 × 224 acquisition matrix, 28 × 5-mm slices, 1-mm slice gap), gradient echo (TR/TE 800/15 ms, 20-degree flip angle, 24 × 18-cm FoV, 384 × 168 acquisition matrix, 2 averages, 28 × 5-mm slices, 1-mm slice gap), sagittal three-dimensional T1-weighted (inversion recovery-prepared spoiled gradient echo TR/TE/TI 7.3/2.9/500 ms, 8-degree flip angle, 330 × 214.5-cm FoV, 256 × 146 acquisition matrix, 100 × 1.8-mm slices) and diffusion tensor MRI (single-shot echo planar imaging with 30 diffusion directions [*b* = 1000 s/mm] and 2 × *b*_0_ acquisitions, TR/TE 7700/82 ms, 24 × 24-cm FoV, 128 × 128 acquisition matrix, 28 × 5-mm slices, 1-mm slice gap).

Patients were asked to return for MRI at 1 year on the same scanner with the same sequences. Throughout the study, the scanner underwent careful daily quality assurance to check that performance remained within tight limits.

### Final diagnosis of stroke and its subtype

The final diagnosis of stroke was determined by an expert panel of stroke physicians, neurologists and neuroradiologists who considered all available clinical and imaging information and decided if the diagnosis was stroke, and, if stroke, then whether it was of the lacunar or cortical subtype according to the Oxfordshire Community Stroke Project classification [14]. Where the clinical syndrome differed from the imaging stroke subtype, as occurs in about 15% of mild stroke cases [15], the imaging subtype was used; where there was no index event visible on imaging (as occurs in about 30% of mild stroke cases [16]), the clinical syndrome was used.

### Image processing

The MRI scans were scored according to the method of Fazekas [17] and for lacunes; perivascular spaces [18]; microbleeds [18]; mineral deposition [19]; the index stroke type; any old infarcts or haemorrhages on presentation and 1-year scans; and any new infarcts, haemorrhages and changes in WMH at 1 year. This scoring was done with a standard pro forma and validated scales by an expert neuroradiologist who was blinded to clinical details [18, 20–24]. The Fazekas scores from baseline and 1-year follow-up images were rated blinded to each other and to clinical information.

The images were also processed using a validated, semi-automated, multispectral image-processing method to determine intracranial, cerebrospinal fluid, whole brain, grey matter, white matter and WMH volumes [25]. In a Bland-Altman analysis measuring WMH volume, this method has a mean difference of 0.38 ml and limits of agreement −2.93 to 3.69 ml [26, 27]. This method fuses combinations of sequences mapped in the colour spectrum and separates tissues according to clusters of signalling. Infarcts (index, old, new during follow-up) were masked from the WMH tissue by manual tracing to avoid contaminating WMH measurements [28].

### Statistical analysis

We used the following hypothesis-driven predictors in simple and multiple linear regression with follow-up WMH volume as an outcome:

- Baseline WMH volume
- Age at initial recruitment
- Final diagnosis of lacunar versus cortical stroke subtype
- Fazekas score (periventricular plus deep white matter) [21]
- Hypertension (blood pressure ≥140/90 mmHg)
- Mean arterial pressure (MAP) (2 × diastolic + systolic blood pressure)/3
- Pulse pressure (PP) (systolic − diastolic blood pressure)
- Diabetes (fasting blood glucose ≥6.1 mmol/L)
- Smoking status (non-smoker for at least 1 year versus current and recent smoker)
- Hyperlipidaemia (total cholesterol >5.0 mmol/L)

We did not choose predictors on the basis of statistical significance, because this can lead to unreliable results [29]. We tested for differences between patients with and without follow-up imaging at 1 year with *t* tests for continuous variables and chi-square tests for categorical variables. We repeated analyses with WMH volume normalised with respect to intracranial volume and obtained nearly identical results (data not presented).

We used our results to conduct sample size calculations for hypothetical studies. The sample size calculations are for an RCT with the hypothesis that the treatment will stabilise WMH volumes and normal progression is 1 ml over the course of 1 year (as seen in our data), with the standard sample size calculation requirements of 80% power and 5% alpha (two-sided), and with a simple comparison of WMH volumes in the control and treatment groups by linear regression. We then changed the analysis plan to include adjustment for the predictors and adjusted the sample size accordingly [30].

Also, we replaced WMH volumes with Fazekas scores as the outcome variable because they are commonly used to assess WMH disease, and then we recalculated the sample sizes. We wanted the Fazekas sample size calculations to be based on the same degree of white matter disease progression as was used in the WMH volume sample sizes. This way the Fazekas sample size estimates could be based on the same underlying change in WMH, even if this change was measured with Fazekas scores rather than WMH volumes. We therefore needed to estimate the change in Fazekas score equivalent to the 1-ml change used in the WMH volume calculations. We used ordinal logistic regression because Fazekas scores are ordinal data, and we estimated the odds ratio (OR) associated with increasing Fazekas score for an increase of 1 ml in WMH volume and used this estimate (OR 1.21, 95% CI 1.16–1.25) in the sample size calculations.

We applied Whitehead’s formula for the logistic regression sample sizes for Fazekas scores. Sample size calculation for logistic regression with baseline adjustment is complex with often untestable assumptions, and it often increases the sample size. One of the assumptions of Whitehead’s formula is that the underlying treatment versus control OR for each baseline category (here, Fazekas group) is the same. This is unlikely to be true in many cases, and Whitehead recommends ‘to try out various scenarios to evaluate the robustness of a proposed design’ [31]. We present below, for interest only, a sample size calculation for a proportional odds model with baseline adjustment, with the caveat that our data would be unlikely to have a constant OR in treatment versus control groups in all baseline categories. If a sample size calculation were required for a real RCT using Fazekas score as the outcome, we would recommend simulation to explore assumptions regarding the OR and the effect of predictors. However, we do not present a full simulation for a sample size calculation, because it is likely to be sensitive to assumptions and selection of pre-specified predictors [32]. Therefore, the results of a simulation for a hypothetical study are unlikely to be widely applicable and may mislead triallists with regard to required sample size. However, the rms package in R [33] has been used for sample size simulation for ordinal logistic regression in the context of stroke, and we recommend this as a template, in particular for the exploration of the proportional odds assumption [34, 35]

Last, we also calculated the sample size for a hypothetical RCT with 1 year follow-up where the treatment group’s Fazekas score would not change but the control group’s would increase as in our dataset—an increase of 0.5 in median Fazekas score. This RCT would use the Wilcoxon rank-sum test to compare median Fazekas scores in the two groups, with the standard assumptions of 80% power and 5% alpha. We considered and rejected using a non-parametric analysis of covariance (ANCOVA) model to allow for baseline Fazekas adjustment in the comparison of medians. There are several non-parametric ANCOVA models available [36–38], but our dataset is not representative of the datasets used to assess the performance of the non-parametric ANCOVA models, because most (183 of 197) patients’ Fazekas scores did not change between baseline and follow-up; that is, the data had many ties, but the non-parametric ANCOVA models were not developed or tested with data where the majority of participants did not change.

We used SAS version 9.3 (SAS Institute, Cary, NC, USA) [39] and R version 2.13.1 [33] software with the add-on package Hmisc [40] for analysis.

## Results

We recruited 264 patients from 10 May 2010 to 24 December 2012, and 190 had imaging data at 1 year. Of the 74 with no 1-year WMH volumes, 33 declined to participate, 24 were unwell, 5 had died, 6 had various other reasons for not participating and 6 had incomplete scans that did not allow calculation of volume data.

Patients who completed follow-up MRI were younger by 5.7 years than patients who did not have another scan. MAP, PP and diastolic and systolic blood pressures were not significantly different between those with and without follow-up MRI (all estimated differences <0.6 mmHg, *p* > 0.73 for all). There was also no significant difference for stroke subtype, hypertension, diabetes, smoking status or hyperlipidaemia (*p* > 0.16 for all). Baseline Fazekas scores tended to be higher in patients without follow-up (*p* = 0.049) (Table 1).

**Table 1:** Estimated differences between patients with (*n* = 190) and without (*n* = 74) follow-up data

Predictor	Summary	Difference between patients with versus without follow-up (95% CI)^a^	*p* Value
Baseline WMH volume, ml, mean ± SD	22.0 ± 24.8	−4.1 (−10.8 to 2.7)	0.23
Age, years, mean ± SD	65.3 ± 11.3	5.7 (−8.8 to −2.6)	0.0004
MAP, mmHg, mean ± SD	102.8 ± 15.4	0.50 (−3.61 to 4.61)	0.81
PP, mmHg, mean ± SD	63 ± 20.7	−0.45 (−5.88 to 4.98)	0.87
Hypertension, *n* (%)	142 (74.7)	8.5% (−3.9 to 20.9%)	0.16
Stroke subtype lacunar, *n* (%)	87 (45.8)	3.9% (−9.4 to 17.2%)	0.57
Smoker (current and recent ex-smokers), *n* (%)	73 (38.4)	−1.9% (−15.1 to 11.4%)	0.78
Diabetes, *n* (%)	21 (11.1)	−1.1% (−9.8 to 7.8%)	0.80
Hyperlipidaemia, *n* (%)	116 (61.1)	0.2% (−12.9 to 13.4%)	0.97
Fazekas score = 0, *n* (%)	8 (4.2)	1.5% (−3.2 to 6.2%)	Overall *p* value = 0.049
Fazekas score = 1, *n* (%)	17 (9.0)	−1.9% (−10.0 to 6.3%)	
Fazekas score = 2, *n* (%)	70 (36.8)	8.5% (−3.9 to 20.8%)	
Fazekas score = 3, *n* (%)	24 (12.6)	3.2% (−5.0 to 11.3%)	
Fazekas score = 4, *n* (%)	29 (15.3)	8.5% (−0.8 to 16.2%)	
Fazekas score = 5, *n* (%)	17 (9.0)	−8.6% (−18.2 to 1.0%)	
Fazekas score = 6, *n* (%)	25 (13.2)	−11.2% (−22.1 to 0.3%)	

*Abbreviations: MAP* Mean arterial pressure, *PP* Pulse pressure, *WMH* White matter hyperintensity

^a^ Estimated difference between patients with and without follow-up WMH volume (difference in means for baseline WMH, age, MAP, PP and difference in percentage for other predictors). A positive number indicates that patients with follow-up had a higher mean or larger proportion

**Fig. 1:**
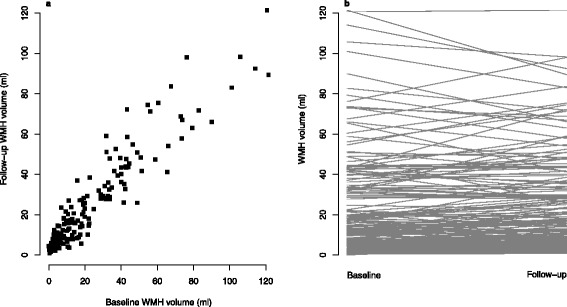
**a** Baseline white matter hyperintensity (WMH) volume versus 1-year follow-up WMH volume. **b** Each *line* represents the change for an individual patient

**Fig. 2:**
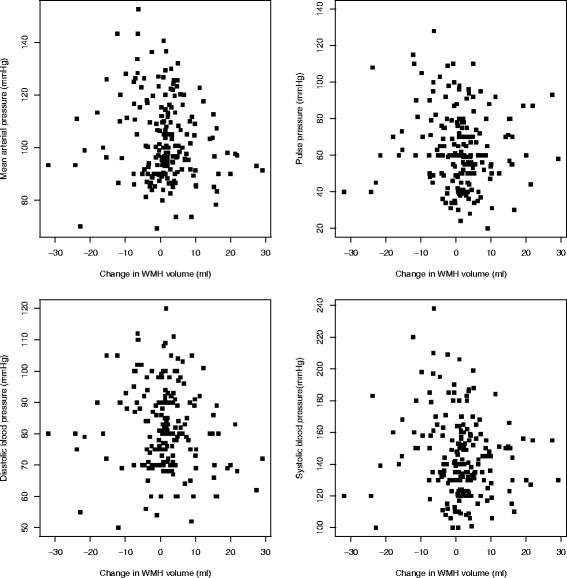
Change in white matter hyperintensity (WMH) (follow-up minus baseline) versus blood pressure

At follow-up, the mean (SD) WMH volume was 23.2 ± 23.3 ml. The mean difference between follow-up and baseline was an increase of 1.27 ml (95% CI 0.06–2.48, *p* = 0.040). A total of 124 patients had an increase (mean increase 5.5 ± 5.8 ml) in WMH volume, whereas 66 had a decrease (mean decrease 6.6 ± 6.9 ml) (Fig. 1). The median Fazekas score at follow-up was 3 (IQR 2–4), a non-significant increase of 0.5 from baseline (2.5; IQR 2–4). There was no statistically significant relationship between blood pressure and WMH change after adjustment (Table 2, Fig. 2).

**Table 2:** Univariate and multivariable linear regression results. Multivariable estimates are adjusted for all other predictors

Predictor	Unadjusted change in WMH volume at 1 year per unit change in predictor (95% CI)	Unadjusted *p* value	Adjusted change in WMH volume at 1 year per unit change in predictor (95% CI)	Adjusted *p* value
Baseline WMH volume, ml	0.88 (0.84 to 0.93)	<0.0001	0.73 (0.65 to 0.80)	<0.0001
Age	0.91 (0.64 to 1.17)	<0.0001	−0.02 (−0.14 to 0.10)	0.75
MAP	−0.11 (−0.33 to 0.11)	0.31	−0.10 (−0.19 to −0.02)	0.013
PP	0.19 (0.035 to 0.35)	0.017	0.03 (−0.03 to 0.10)	0.34
Hypertension	4.89 (−2.77 to 12.54)	0.21	−1.40 (−4.06 to 1.26)	0.30
Fazekas score	11.54 (10.43 to 12.65)	<0.0001	2.93 (1.76 to 4.10)	<0.0001
Stroke subtype	0.88 (−5.83 to 7.58)	0.80	−0.85 (−3.05 to 1.34)	0.44
Smoking status	−2.30 (−9.16 to 4.56)	0.51	0.17 (−2.24 to 2.57)	0.89
Diabetes	10.36 (−0.19 to 20.90)	0.054	−1.98 (−5.51 to 1.54)	0.27
Hyperlipidaemia	−0.51 (−7.36 to 6.34)	0.88	−1.10 (−3.40 to 1.19)	0.34

*Abbreviations: MAP* Mean arterial pressure, *PP* Pulse pressure, *WMH* White matter hyperintensity

We expected and checked for collinearity with variance inflation factors, because MAP, PP and diagnosis of hypertension are all ways of assessing blood pressure, and Fazekas score and baseline WMH volume are both ways of assessing WMH burden. The blood pressure variance inflation factors were 1.4, 1.6 and 1.2, respectively, for MAP, PP and diagnosis of hypertension, indicating that they are not a source of collinearity. However, Fazekas score and baseline WMH volume have variance inflation factors of 3.4 and 3.1, respectively. This may or may not indicate an acceptable degree of collinearity to researchers, so the results should be interpreted cautiously, though it should be noted that dropping Fazekas score from the model gives similar results.

The predictor that explained most of the variation in follow-up WMH volume was baseline WMH volume in both univariate and multivariable analyses (Table 2, Fig. 1a). Fazekas score was also related. Uncertainty in the univariate analyses (evidenced by wide CIs) and changes of directions for some predictors (e.g. diabetes) were observed. Individual changes in WMH volume were varied, and WMH decreased in some patients, stayed the same in some, and increased in others, reflected in the wide SD. MAP was negatively associated with WMH progression in adjusted analysis (Table 2), as suggested in Fig. 1, where there are more patients with higher starting blood pressure with decreasing WMH.

The sample size calculations for WMH volume were based on an RCT in which the treatment was hypothesised to stabilise WMH volume as at trial entry by preventing progression of WMH. In the control group, WMH would increase, as seen in our observational data at approximately by 1 ml per year, and the WMH volume SD at follow-up would be 23.3 ml. We then adjusted the sample size for inclusion of predictors in the analysis. The *R* ^2^ values are taken from regression models with follow-up WMH volume as the outcome, and they are used to calculate the reduction in sample size that would happen by including adjustment for predictors by reducing the unexplained variability in outcome. We then recalculated the sample size for a 2-ml difference between active and control groups and for 2 years of follow-up, assuming the same per-year increase in WMH. The sample size calculations for Fazekas score are based on our baseline and follow-up data percentages of patients in each category.

If the RCT had allowed for 2 years of follow-up and with the same assumptions so that the control group WMH volume would increase by 2 ml, then the sample size per group would be 2128 (4256 in total). Adjustment for all predictors reduces this to 205 per group (410 in total) before allowing for dropouts (Table 3).

**Table 3:** Sample sizes for hypothetical randomised controlled trials with different analysis plans/outcome variable

Predictors included in RCT analysis	Outcome variable at 1-year follow-up^a^	Sample size (*n* per group)	Total sample size	Allowance for 20% dropout
Treatment group only (assumed difference 1 ml)	WMH volume	8508	17,016	21,270
Treatment group + baseline WMH volume (*R* ^2^ = 0.88)	WMH volume	987	1974	2468
Treatment group + age (*R* ^2^ = 0.19)	WMH volume	6872	13,744	17,180
Treatment group + baseline Fazekas score (*R* ^2^ = 0.69)	WMH volume	2628	5256	6570
Treatment group + all predictors used in multivariable analysis (*R* ^2^ = 0.90)	WMH volume	821	1642	2054
Treatment group + all predictors used in multivariable analysis (*R* ^2^ = 0.90) and 2-year follow-up	WMH volume	205	410	642^b^
Treatment group only (assumed OR 1.2)	Fazekas score	1499	2998	3748
Treatment group only (assumed OR 1.25, upper 95% CI)	Fazekas score	1001	2002	2504
Treatment group only (assumed OR 1.16, lower 95% CI)	Fazekas score	2262	4524	5656
Treatment group + baseline Fazekas (multiplier^c^ 5.04)	Fazekas score	7554	15,108	18,886
Treatment group + baseline hypertension (multiplier^c^ 1.00057)	Fazekas score	1500	3000	3750
Treatment group + baseline age (multiplier^c^ 1.012)	Fazekas score	1515	3030	3788
Treatment group only (median increase 0.5)	Fazekas score	87,216	174,432	218,040

*RCT* Randomised controlled trial, *WMH* White matter hyperintensity

^a^ Except for second multivariate sample size estimate, where 2-year follow-up was used

^b^ A 20% dropout per year was used for the 2-year follow-up (i.e., a total dropout rate of 36%)

^c^ The multiplier is the number by which the base sample size must be multiplied to allow for the inclusion of an additional predictor in the analysis without loss of power; the base sample size is that for an analysis with Fazekas score as outcome and treatment group as the only predictor

If Fazekas score were the outcome variable, then sample sizes would increase by a factor of 5.04 between not adjusting and adjusting for baseline Fazekas score, assuming a constant OR for treatment versus control in all baseline Fazekas categories. This assumption is not likely to be true and would need to be explored with a simulation study. Other predictors would inflate the sample size less, given the same assumption (Table 3). The large sample size for the comparison of medians is a result of most patients’ Fazekas scores not changing over the course of 1 year.

## Discussion

WMH volumes are linked to cognitive decline, stroke, dementia and death. Although their causes have not yet been fully ascertained, treatment of risk factors such as blood pressure may limit their progression. These interventions are being tested in ongoing trials. We studied a well-defined patient sample with mild non-disabling ischaemic stroke, a group likely to be of interest to researchers in stroke and dementia. The WMH volumes were measured carefully with a well-developed methodology. In our observational cohort of patients followed for 1 year, there was wide variation in WMH volume change from patient to patient, making prediction for individual patients difficult. Roughly one-third of our sample showed a decrease in WMH, and two-thirds showed an increase. The extent of change also varied widely, with a mean change of 1.27 ml but a range from −32.0 ml to +29.1 ml. The predictor that explained most of the variation in WMH progression was WMH volume or Fazekas score at presentation.

Previous estimations of average rates of progression of WMH were similar to ours at about 1 ml per year, but they did not seem to identify the relatively large proportion whose WMH decreased [8, 9]. This may reflect the fact that other populations, such as community-dwelling older subjects as in the Leukoaraiosis and Disability Study [10] or the Austrian Stroke Prevention Study [41], may be less variable than patients presenting with an acute disease such as stroke.

Researchers seeking to undertake RCTs of treatment to prevent WMH progression need to be aware that the variation in WMH volumes, at least in stroke patients, can lead to prohibitively large sample sizes unless an efficient study design is used. Fortunately, making RCTs more efficient need not be difficult. Analyses that use a baseline measurement as a predictor are known to be more efficient than studies comparing follow-up measurements only, or even those that use difference between baseline and follow-up [42–44]. Our calculations for hypothetical studies show that it is possible to reduce the required sample size by more than 90% by including some routinely collected data and a baseline measurement. Increasing duration of follow-up to 2 years also helps reduce sample size but increases cost per patient substantially as well as the number of dropouts.

However, a trial of 1642 patients (followed over 1 year, 2054 allowing for dropouts) is a massive undertaking, especially if all must have baseline and follow-up MRI and all images must be analysed for WMH volume, which has to be done centrally by trained operators, including careful exclusion of the index and any old stroke lesions. Of note, 74 (28%) of 264 in our cohort did not have MRI at 1 year. Failure to have repeat MRI is not uncommon in observational studies and trials.

We did not allow for a range of dropout rates, and we present data for only a 20% per year dropout rate. This (and other rates) of course increases the required sample size. Here, just under one-third (28%) did not have 1-year MRI. Although this might have been different in an RCT (rather than an observational study), we have no evidence that patients are more likely to comply with imaging in trials. Moreover, 29 (39%) of 74 patients who dropped out did so for reasons of health, which is likely to be a source of bias; it seems reasonable to suppose that people with worse general health have less healthy white matter and hence more WMH progression. This bias would have a greater effect in studies with longer follow-up, because dropout is generally greater in long-term studies. The attractive sample size estimate for the 2-year study needs to be balanced against this bias. Statistical methods for dealing with missing data generally assume that the data are missing at random. Although the methodology of dealing with data that are not missing at random is developing, and although it is possible that data more readily obtainable at follow-up could be used in multiple imputation, any results would need to be interpreted very cautiously. In particular, the sensitivity of results to assumptions about the relationship of missingness and observed data would require careful exploration. Additionally, the longer the follow-up, the greater the risk of genuine changes in MRI scanner performance or in scanner replacement, which could inflate variance and further alter the detection of WMH. Finally, 6 (8%) of the 74 subjects without follow-up MRI data actually had the scan, but their data were unsuitable for WMH volume measurement because of patient movement within the scanner or completing only part of the scan.

Over 10% (33 of 264) of patients (45% of the 74 who did not have 1-year MRI) did not have follow-up WMH volume data because they found the first MRI scan unpleasant. Although careful screening of patients with claustrophobia and other contraindications is essential, it is not possible to predict which patients will decline follow-up because it involves MRI.

We know that the patients with follow-up were younger and had lower Fazekas scores than the entire baseline group. Although we adjusted for age, Fazekas score and other predictors, the baseline and follow-up groups may differ in other ways that we have not been able to account for.

We have not allowed for the effects of between-scanner variation on measurement of WMH volume, but large trials (even to get the minimum number of 642 patients calculated here) require multicentre participation, which inevitably requires use of different scanners and introduces between-scanner variation. Whether the Fazekas score (or other visual scores) are less susceptible to between-scanner variation is not known and would be an obvious target for testing to inform future trials in stroke and dementia.

Fazekas scores are more easily measured than WMH volumes. However, calculation of the sample size for scales such as the Fazekas scale is more complex and requires data that may not be readily available. The results shown in Table 3 suggest that the sample size is sensitive to small changes in the assumed OR—a small decrease in OR of just 0.04 caused the sample size to more than double, and a small increase in OR of 0.05 more than halved the number. Therefore, researchers should use the most precise estimates available to ensure than any ordinal logistic regression analysis is suitably powered. Also, sample size may counterintuitively increase when estimates are adjusted for baseline covariates [45]. Even so, it is recommended that researchers use adjustment in logistic regression both to balance the trial groups for important outcome predictors and to have estimates that are more readily applicable to individual patients [34]. Researchers need to undertake a simulation study to explore assumptions and the sensitivity of the estimated sample size to those assumptions. A compromise for the significant organisational effort, cost and ‘noise’ involved in assessing baseline WMH volume that captures the similar prognostic information might be to use the baseline Fazekas score and adjust for covariates and use WMH volumes as outcome. It is also important to tie WMH to a relevant clinical outcome such as cognition, recurrent stroke, other vascular events or death, and possibly a measure of mobility, to capture the physical effects of small vessel disease.

In our statistical model, we used raw WMH volumes and checked model performance by examining residual behaviour and linearity assumptions. Some aspects of model performance were improved by using the logarithm of the WMH volumes rather than the raw data. The greatest improvements occurred when we used logarithms for both baseline and follow-up data. In our opinion, the improvement in model performance with logged data was not great, so we chose to present results for the raw data for ease of interpretation. However, we recognize that this is a subjective choice and that others might choose differently. Unsurprisingly, the models with raw and logged data all revealed baseline WMH to be positively related to follow-up WMH, with high *R* ^2^ values of 0.90 for raw baseline and follow-up and 0.87 for logged baseline and follow-up. Therefore, the reduction in sample size would be similar, regardless of whether logged or raw data were used.

Although our sample size for the comparison of medians gave an unachievable sample size (174,432 before allowing for dropouts), this does not mean that analysis with medians in other contexts is unfeasible. Researchers wishing to use medians could explore the non-parametric ANCOVA models with simulation studies, not only to estimate sample size but also to test the suitability of the ANCOVA models for the kind of data they are likely to obtain. This is beyond the scope of this paper, but it could be a subject for further research.

We show, in a fairly typical population of patients presenting to a regional hospital with non-disabling stroke and managed according to current UK guidelines with anti-hypertensives, statins, antiplatelet drugs and lifestyle advice, that WMH may decrease, stay the same and increase over the course of 1 year after stroke. Baseline MAP was inversely associated with WMH volume in multivariate analyses. This is counterintuitive, but it may have occurred if patients with higher initial MAP were diagnosed when presenting to the stroke clinic and were started on anti-hypertensive medication, or if they already had a diagnosis of hypertension but were not taking their prescribed anti-hypertensive medications and were motivated by their stroke to take their medications, leading to better blood pressure control, which may have contributed to reducing the development of WMH. In general, WMH are assumed only to increase in size, but the fact that they may decrease as shown here (in 35%) adds to the variation in follow-up volumes, thus inflating sample sizes, and indicates that they do not consist only of permanently damaged tissue. However, it would seem that WMHs are much more dynamic, at least in the year after mild stroke, than previously thought. Reasons for this dynamic WMH change, as well as the primary mechanisms underlying WMH, should be determined urgently.

## Conclusions

Sample sizes for RCTs using WMH as an outcome can vary enormously according to measurement of WMH, adjustment for predictors, and method of analysis. Smaller sample sizes will be obtained in studies that use WMH volumes rather than Fazekas scores, but measuring volumes is resource-intensive. Dropout rates and scanner drift could impact results. Triallists undertaking studies using WMH as an outcome need to consider their study design and analysis plan carefully to maximise efficiency.

## Abbreviations

ANCOVA: Analysis of covariance
BP: blood pressure
CI: Confidence interval
FoV: Field of view
IQR: interquartile range
MAP: Mean arterial pressure
MRI: Magnetic resonance imaging
OCSP: Oxfordshire Community Stroke Project
OR: Odds ratio
PP: Pulse pressure
RCT: Randomised controlled trial
SD: Standard deviation
TE: Echo time
TI: Inversion time
TR: Repetition time
WMH: White matter hyperintensity
